# Knowledge, Attitudes, and Practices Regarding Herpes Zoster and Its Vaccine Among Primary Health Care Attendees in the United Arab Emirates: A Cross-Sectional Study

**DOI:** 10.7759/cureus.107442

**Published:** 2026-04-21

**Authors:** Fatima A Almeleh, Shaikha Al Hayayi, Amnah Alsalami

**Affiliations:** 1 Primary Health Care, Emirates Health Services, Dubai, ARE; 2 Family Medicine, Emirates Health Services, Fujairah, ARE

**Keywords:** aged, attitudes, health knowledge, herpes zoster, practice, primary health care, united arab emirates, vaccination

## Abstract

Background

Herpes zoster (HZ) is a reactivation of the latent varicella-zoster virus, with incidence increasing in older adults. Despite the availability of an effective vaccine, uptake remains low globally and in the United Arab Emirates (UAE).

Objectives

The objective of the study was to assess knowledge, attitudes, and practices (KAP) regarding HZ and its vaccine among primary health care (PHC) attendees in the UAE, and to identify demographic factors associated with knowledge levels.

Methods

A cross-sectional study was conducted from March to May 2025 in primary health care (PHC) centers within Emirates Health Services (EHS) across six emirates of the United Arab Emirates. Adults aged ≥18 years were recruited using convenience sampling. Data were collected via a validated, adapted questionnaire covering sociodemographic characteristics and knowledge, attitudes, and practices regarding HZ and its vaccine. Associations were analyzed using chi-square tests.

Results

Of 389 participants, 268 (68.9%) were female, and 265 (68.1%) were aged 18-49 years. Knowledge levels were high in 67 (17.2%), moderate in 197 (50.6%), and low in 125 (32.1%) participants. Higher knowledge was significantly associated with female gender, younger age, higher education, health insurance, and prior chickenpox history (all p<0.05). Most respondents, 294 (76.0%), were interested in learning about HZ, 317 (81.5%) were interested in prevention strategies, and 287 (73.7%) reported willingness to vaccinate if recommended by a physician. Vaccine cost was reported as likely or extremely likely to influence decision-making by 140 (35.9%) participants. Overall, 6.4% (n = 25) of participants reported having already received the herpes zoster vaccine. The most common reasons for hesitancy included fear of side effects (77; 19.8%) and low perceived risk (34; 8.7%).

Conclusion

Knowledge of herpes zoster among PHC attendees was moderate, while attitudes toward vaccination were generally favorable, particularly when supported by a physician's recommendation. Targeted educational interventions, strengthened physician engagement, and addressing cost concerns may help improve vaccine uptake in the UAE.

## Introduction

Varicella zoster virus (VZV) is a double-stranded DNA herpesvirus that can cause a primary infection called varicella (chicken pox) and a latent infection called herpes zoster (HZ), also known as shingles. Varicella is a widespread, highly contagious infection that occurs mainly in children, especially in late winter and spring. It manifests as a vesicular rash at different developmental stages, mostly affecting the scalp, face, and trunk [1]. After primary infection or vaccination, VZV remains latent in dorsal root ganglia and may reactivate as herpes zoster, causing a painful dermatomal rash [2]. The incidence rates of HZ range from 5.49 to 8.67 per 1,000 people in North America, 5.77 to 9.85 per 1,000 people in Europe, and 2.9 to 19.5 per 1000 people in the Asia-Pacific region [3]. In the United States, the incidence rate, which varies with age, ranges from two to nine cases per 1000 people [4]. It is anticipated that the incidence of HZ will increase due to an aging population, suggesting a rise in the HZ burden [3]. The main risk factor for HZ is age, commonly seen in adults ≥50 years old due to the age-related decrease in cell-mediated immunity [1,3]. Roughly 20% of cases present in people aged 50-59, and 40% in those aged 60 and above. Other risk factors include female sex, Caucasian ethnicity, being immunocompromised (due to HIV, cancer, autoimmune disorders, or transplant status), and having comorbidities such as chronic lung or kidney disease, diabetes, and depression [1,3,4].

Herpes zoster can adversely impact the quality of life in older adults, causing serious neurological or ocular complications. The most common neurological complication is postherpetic neuralgia (PHN), where pain persists for months to years after the resolution of the rash. Other neurological complications include cranial nerve palsies, zoster paresis, ischemic stroke, meningoencephalitis, myelopathy, and cerebellitis. HZ can also lead to vision loss, particularly with cranial nerve involvement, such as in herpes zoster ophthalmicus [5]. It is recommended, by the US Centers for Disease Control (CDC) and the Advisory Committee on Immunization Practices (ACIP), that adults aged 50 years and older, as well as adults aged 19 years and older at high risk, receive two doses of the recombinant zoster vaccine (RZV) for the prevention of HZ and related complications [6]. A systematic review and network meta-analysis showed the RZV is 94% effective compared to placebo in preventing HZ [7]. Research has demonstrated that the zoster vaccine is an important public health tool, not only reducing the incidence of HZ but also proving to be cost-effective by decreasing the long-term healthcare burden associated with the disease [8].

Studies on public knowledge of HZ and its vaccine are lacking in the United Arab Emirates (UAE), mirroring a wider research gap on HZ in the Middle East and North Africa (MENA) region. The only study conducted in the UAE was in Sharjah in 2019 with 420 participants. The study concluded that although 64.3% of participants had heard of HZ, only 14.8% were aware of the HZ vaccine, and 96.7% had not received the vaccine [9]. Regionally, a cross-sectional study with 1883 participants conducted in Saudi Arabia in 2023 looked at the level of HZ knowledge. The study showed that 78.9% of participants had heard of HZ, but only 3.1% had adequate knowledge about the vaccine [10]. Evidently, there is a substantial gap between knowledge of HZ and its vaccine and, consequently, vaccine administration. Since 2023, the UAE Ministry of Health and Prevention has been offering the HZ vaccine as part of the national adult vaccination program for adults aged 50 and above as an initiative to prevent HZ and its complications, raise public awareness, and educate healthcare professionals [11]. This initiative aims to improve community awareness of HZ in the UAE and increase vaccine uptake, thereby supporting national immunization objectives.

This study aimed to: (1) assess knowledge, attitudes, and practices toward HZ and its vaccine among primary healthcare center (PHC) attendees in Emirates Health Services (EHS) facilities; (2) examine associations between sociodemographic factors and knowledge levels regarding HZ and its vaccine; and (3) descriptively explore awareness and uptake of the HZ vaccine in the context of the national adult vaccination program.

The findings are primarily representative of PHC attendees within EHS facilities and may not be fully generalizable to the broader UAE population.

## Materials and methods

Study design and setting 

This multi-center, cross-sectional study was conducted across 61 primary healthcare (PHC) centers within Emirates Health Services (EHS) in six emirates of the United Arab Emirates (excluding Abu Dhabi, which is served by a separate healthcare system) between March and May 2025. All EHS PHC centers offering herpes zoster vaccination services were included, with no selection of centers performed. Participants’ reported emirate of residence spanned all seven emirates, including Abu Dhabi.

A non-probability, convenience sampling approach was adopted, following the methodology of a previous UAE-based investigation [9]. Sampling was not proportional by emirate, and site-level enrolment volumes reflected routine clinic throughput and scheduling rather than emirate population size. Adults aged 18 years or older who attended any of the participating centers during the study period were eligible for inclusion. Based on a 95% confidence level (Z = 1.96), an assumed 50% prevalence (p = 0.50), and a 5% margin of error (e = 0.05), a minimum sample size of 385 was calculated. To accommodate potential non-responses and incomplete questionnaires, we inflated this target by 10-15%, aiming to recruit approximately 424 participants.

Study participants 

The study included individuals aged 18 years or older who could understand Arabic or English and who provided electronic informed consent. Those younger than 18 years, unable to provide electronic consent, or lacking proficiency in either study language were excluded. All eligible adults attending the selected healthcare centers during the study period were invited to participate.

Data collection tools and process

The study's questionnaire (Appendix 1) was adapted, with permission from the corresponding author, from a previously validated instrument developed by Al-Khalidi et al. [9]. The original tool demonstrated face validity and content reliability, having undergone expert review by a language specialist and a biostatistician, and was piloted on 20 participants for clarity and cultural appropriateness prior to its initial use. In that validation study, the instrument showed satisfactory internal consistency, with Cronbach’s alpha values of 0.82 for the knowledge section, 0.79 for the attitudes section, and 0.81 for the practices section. In the present study, the questionnaire was modified to align with the objectives and context of primary health care attendees in the UAE. Modifications included removing items not relevant to the target population, rephrasing selected questions for clarity, and adding new items to capture additional variables of interest. For example, items unrelated to herpes zoster vaccination were removed, and additional questions were added to assess awareness of herpes zoster risk factors and vaccine eligibility. The adapted questionnaire retained the original structure and key domains of knowledge, attitudes, and practices regarding herpes zoster and its vaccine. To ensure content validity after adaptation, the instrument was reviewed by a consulting biostatistician and piloted on a small group of primary health care attendees to assess clarity, comprehension, and cultural appropriateness. The internal consistency of the adapted version was not reassessed in this study.

After providing electronic informed consent through the online survey platform, participants received a self-administered, closed-ended questionnaire through the Emirates Health Services Data Hub System, an authorized platform for secure email and SMS survey distribution. The online survey link was distributed to eligible participants via an automated SMS and email platform managed by the Emirates Health Services (EHS) Data Hub after they attended PHC centers during the study period, ensuring secure data handling and response monitoring. The EHS Data Hub system restricts duplicate submissions, ensuring that each participant can respond only once.

The 27-item instrument, adapted from a Sharjah University study [9], was available in both English and Arabic. In the present study, both language versions were used based on participant preference. The questionnaire was reviewed by a language specialist to ensure clarity, consistency, and cultural appropriateness. The instrument covered four domains: demographic characteristics (10 items), knowledge of herpes zoster and its vaccine (10 items), attitudes toward the disease and vaccination (five Likert-scale items), and vaccination practices ( items). Responses to the question on reasons for herpes zoster vaccine uptake or refusal were categorized into two groups: acceptance (willingness or prior vaccination) and hesitancy (specific refusal reasons). Percentages for each category were calculated based on the total number of respondents, with multi-response options allowed. A consulting biostatistician reviewed the questionnaire to confirm content validity and analytic suitability before field deployment. For each knowledge question, a correct answer was given a point. Responses of ‘don’t know’ were treated as incorrect. Knowledge scores were summed and classified using modified Bloom’s cut-offs: High (≥80%), Moderate (60-79%), and Low (<60%). As all questionnaire items were mandatory in the online survey platform, no missing or incomplete responses were recorded.

Pilot study 

We conducted a pilot study with 20 convenience-sampled individuals recruited from primary healthcare center attendees who met the inclusion criteria to evaluate the questionnaire’s clarity, cultural appropriateness, and feasibility. Based on participant feedback, minor wording adjustments were made to improve comprehension. Data from the pilot cohort were excluded from the final analysis

Ethical considerations 

Ethical approval was obtained from the Ministry of Health and Prevention Research Ethics Committee before study initiation (Approval Reference No: MOHAP/DXB-REC/ M.M.A /No. 49/ 2025). Electronic informed consent was obtained from all participants before survey completion. Participation was entirely voluntary, and no personally identifiable information was collected. All data were anonymized and stored on encrypted servers to maintain participant confidentiality and adhere to ethical research standards.

Statistical analysis 

The dataset was coded, cleaned, and analyzed using IBM SPSS Statistics for Windows, Version 25 (IBM Corp., Armonk, USA). Descriptive statistics (frequencies, percentages, means, and standard deviations) described participant characteristics and primary outcomes. Inferential tests, including Chi-square tests, were used to assess associations between categorical variables. Findings were presented via bar graphs, pie charts, and tables to facilitate clear interpretation. A p-value of less than 0.05 was considered statistically significant.

## Results

Table 1 summarizes the demographic and clinical characteristics of the participants (n = 389). The majority were female (68.9%) and aged 18-49 years (68.1%). Most participants were employed (61.4%), while smaller proportions were unemployed (28.0%), retired (8.2%), or self-employed (2.3%).

**Table 1:** Demographic and clinical characteristics of participants (n = 389) Percentages are calculated from the total sample (n = 389). For chronic diseases, percentages sum to >100% because participants could select multiple conditions. Abbreviations: COPD, chronic obstructive pulmonary disease; GCC, Gulf Cooperation Council; CVD, cardiovascular disease.

Demographic	Category	n (%)
Gender	Male	121 (31.1)
	Female	268 (68.9)
Age (years)	18–49	265 (68.1)
	50–59	108 (27.8)
	60 or above	16 (4.1)
Working Status	Employed	239 (61.4)
	Unemployed	109 (28.0)
	Self-employed	9 (2.3)
	Retired	32 (8.2)
Nationality	Emirati	277 (71.2)
	Arab	77 (19.8)
	GCC	10 (2.6)
	Other nationality	25 (6.4)
Insurance	Yes	265 (68.1)
	No	124 (31.9)
Education Level	Less than high school	10 (2.6)
	High school graduate	135 (34.7)
	University/college graduate	195 (50.1)
	Postgraduate degree (Master’s, Ph.D., etc.)	49 (12.6)
Chickenpox History	Yes	216 (55.5)
	No	137 (35.2)
	Don’t know	36 (9.3)
Chronic Diseases	COPD	12 (3.1)
	Hypertension	53 (13.6)
	Thyroid disease	31 (8.0)
	Diabetes	43 (11.1)
	Autoimmune diseases (lupus, rheumatic)	7 (1.8)
	Cancer	3 (0.8)
	CVD	4 (1.0)
	Kidney disease	4 (1.0)
	Don’t have any	268 (68.9)

Emirati nationals comprised the majority (71.2%), followed by Arabs (19.8%), other nationalities (6.4%), and Gulf Cooperation Council (GCC) nationals (2.6%). Most participants had health insurance (68.1%). In terms of education, approximately half were university or college graduates (50.1%), with smaller proportions holding postgraduate degrees (12.6%), high school education (34.7%), or less than high school education (2.6%).

More than half (55.5%) reported a history of chickenpox infection, while 35.2% had no history and 9.3% were unsure. Most participants reported no chronic disease (68.9%); among those with comorbidities, the most common conditions were hypertension (13.6%), diabetes (11.1%), and thyroid disorders (8.0%).

Table 2 presents the distribution of participants by emirate of residence. The largest proportions of participants were from Sharjah (21.6%), Fujairah (19.3%), and Ras Al Khaimah (17.0%), while smaller proportions were from Dubai (15.2%), Ajman (12.3%), Abu Dhabi (9.3%), and Umm Al Quwain (5.4%). This distribution reflects variation in recruitment across emirates and is not proportional to population size.

**Table 2:** Distribution of study participants by emirate of residence (n = 389) Values are presented as number (%). Recruitment volumes reflected clinic throughput and scheduling at participating primary health care centers; sampling was not proportional by emirate.

Emirate	n	%
Abu Dhabi	36	9.3
Dubai	59	15.2
Sharjah	84	21.6
Ajman	48	12.3
Umm Al Quwain	21	5.4
Ras Al Khaimah	66	17.0
Fujairah	75	19.3
Total	389	100.0

**Figure 1:**
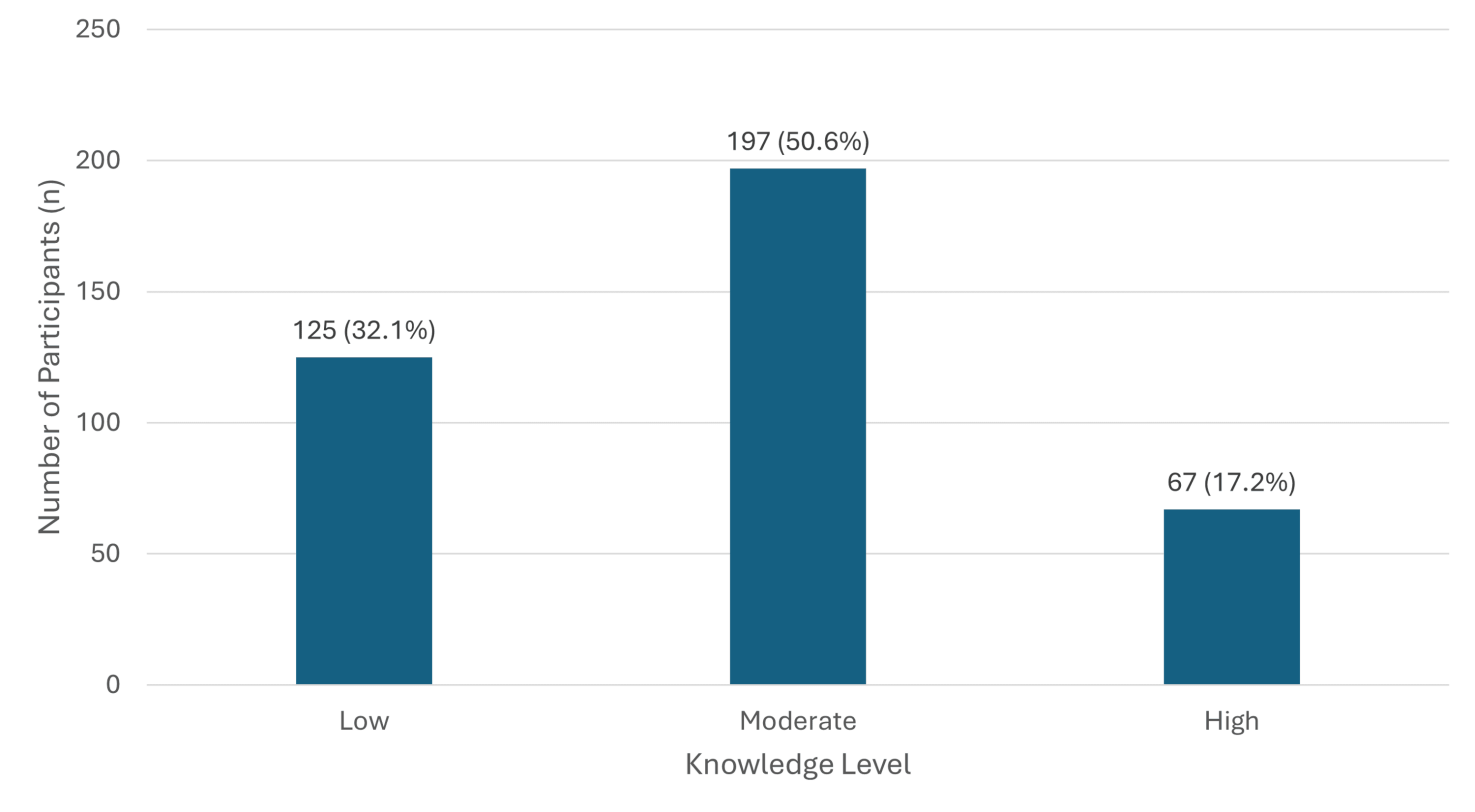
Knowledge level of participants regarding herpes zoster and its vaccination (n = 389) Values are presented as n (%)

Regarding knowledge levels, 32.1% (n = 125) of participants had low knowledge, 50.6% (n = 197) had moderate knowledge, and only 17.2% (n = 67) demonstrated high knowledge of HZ (Figure 1). The mean knowledge score was 3.94 ± 2.23 (SD) out of a maximum score of 10.

Knowledge levels differed significantly across demographic groups. Males were more likely to have low knowledge (61; 50.4%) compared to females (64; 23.9%), while a higher proportion of females demonstrated moderate knowledge (154; 57.5%) than males (43; 35.5%) (p < 0.001). Participants aged 50-59 years had the highest proportion of low knowledge (57; 52.8%), whereas those aged 18-49 years more commonly demonstrated moderate knowledge (155; 58.5%) (p < 0.001).

Employment status was not significantly associated with knowledge level (p = 0.116), although employed participants showed a higher proportion of moderate knowledge (120; 50.2%). Nationality was significantly associated with knowledge (p = 0.017); Emiratis had the highest proportion in the moderate knowledge category (139; 50.2%), while participants of other nationalities and GCC backgrounds had higher proportions in the high knowledge category (9; 36.0%, and 4; 40.0%, respectively).

Health insurance was also significantly associated with knowledge level (p = 0.015), with uninsured participants showing a higher proportion of moderate knowledge (76; 61.3%) compared to insured participants (121; 45.7%). Educational level was strongly associated with knowledge (p < 0.001). Participants with postgraduate education had the highest proportion of moderate knowledge (33; 67.3%) and the lowest proportion of high knowledge (5; 10.2%), while those with less than high school education had the highest proportion of high knowledge (5; 50.0%); this likely reflects the small subgroup size (n = 10) and should be interpreted with caution.

Participants with a history of chickenpox had a higher proportion of moderate knowledge (125; 57.9%) compared to those without (47; 34.3%) or those who were unsure (25; 69.4%) (p < 0.001) (Table 3).

**Table 3:** Association between demographic characteristics and knowledge levels regarding herpes zoster and its vaccination (n=389) P-values were calculated using the Chi-square test for independence. Percentages for subgroups with small denominators (n < 20) are statistically unstable and should be interpreted with caution. Abbreviations: GCC, Gulf Cooperation Council.

Demographic	Category	Low Knowledge Level (n = 125) n (%)	Moderate Knowledge Level (n = 197) n (%)	High Knowledge Level (n = 67) n (%)	P-value	Chi-square test values
Gender	Male	61 (50.4)	43 (35.5)	17 (14.0)	<0.001	27.2
	Female	64 (23.9)	154 (57.5)	50 (18.7)		
Age (years)	18–49	64 (24.2)	155 (58.5)	46 (17.4)	<0.001	31.5
	50–59	57 (52.8)	34 (31.5)	17 (15.7)		
	60 or above	4 (25.0)	8 (50.0)	4 (25.0)		
Working Status	Employed	87 (36.4)	120 (50.2)	32 (13.4)	0.116	10.2
	Self-employed	3 (33.3)	3 (33.3)	3 (33.3)		
	Unemployed	27 (24.8)	57 (52.3)	25 (22.9)		
	Retired	8 (25.0)	17 (53.1)	7 (21.9)		
Nationality	Emirati	99 (35.7)	139 (50.2)	39 (14.1)	0.017	15.4
	Arab	19 (24.7)	43 (55.8)	15 (19.5)		
	GCC	1 (10.0)	5 (50.0)	4 (40.0)		
	Other nationality	6 (24.0)	10 (40.0)	9 (36.0)		
Insurance	Yes	95 (35.8)	121 (45.7)	49 (18.5)	0.015	8.4
	No	30 (24.2)	76 (61.3)	18 (14.5)		
Education Level	Postgraduate degree (Master’s, Ph.D., etc.)	11 (22.4)	33 (67.3)	5 (10.2)	<0.001	32.4
	University/college graduate	48 (24.6)	111 (56.9)	36 (18.5)		
	High school graduate	62 (45.9)	52 (38.5)	21 (15.6)		
	Less than high school	4 (40.0)	1 (10.0)	5 (50.0)		
Chickenpox History	Yes	52 (24.1)	125 (57.9)	39 (18.1)	<0.001	29.9
	No	66 (48.2)	47 (34.3)	24 (17.5)		
	Don’t know	7 (19.4)	25 (69.4)	4 (11.1)		

**Figure 2:**
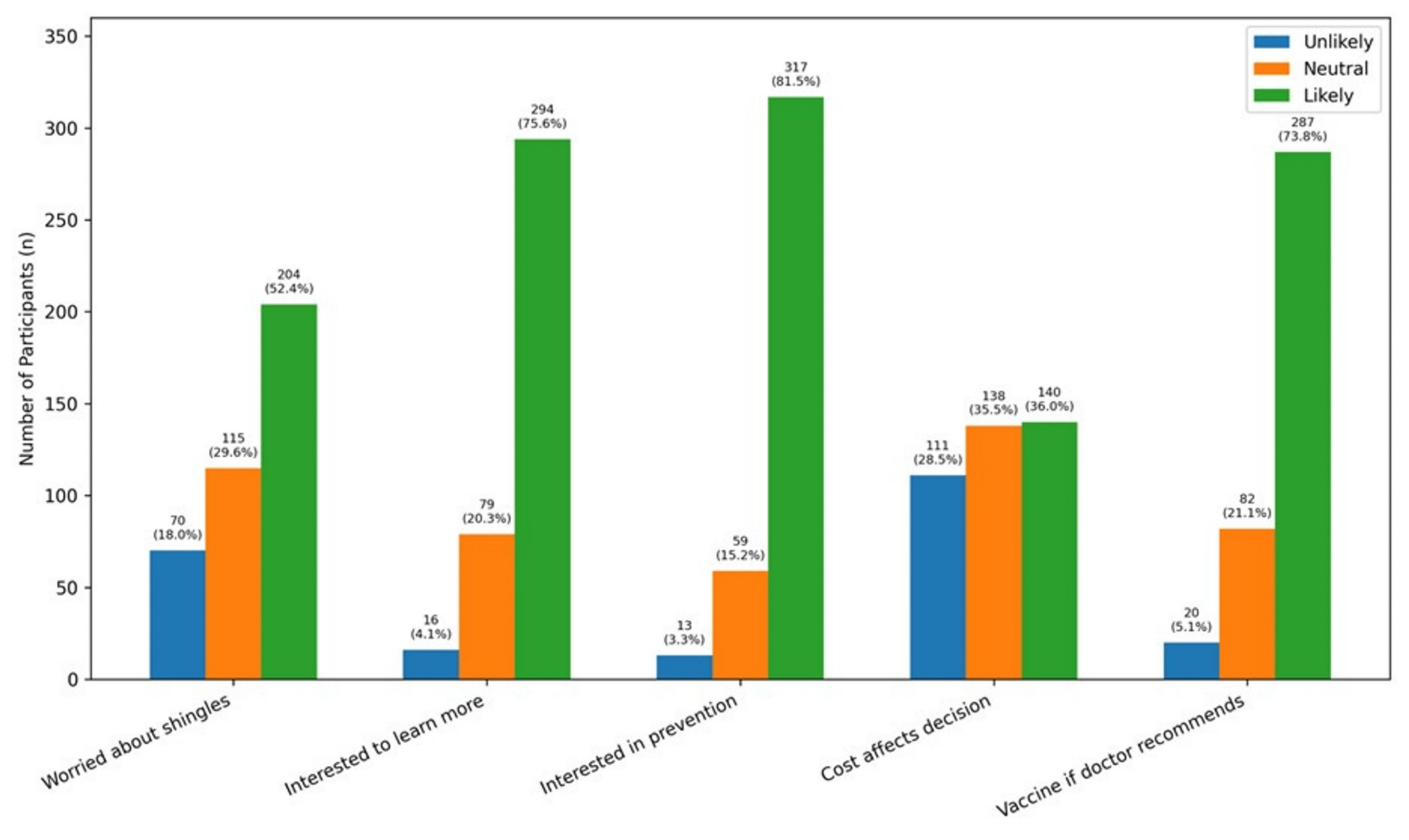
Attitudes of participants regarding herpes zoster and its vaccination (n = 389) Values are presented as n (%). Response categories were collapsed into three groups: unlikely (extremely unlikely and unlikely), neutral, and likely (likely and extremely likely).

Regarding attitudes, participants demonstrated a strong interest in learning about HZ and its prevention. Overall, 76.0% (n = 294) were likely or extremely likely to seek more information about the disease, and 81.5% (n = 317) expressed interest in prevention strategies. More than half (52.5%; n = 204) reported concern about developing HZ, while 29.6% (n = 115) were neutral. Cost showed a moderate influence on vaccination decisions, with 35.9% (n = 140) indicating it would likely or extremely likely affect their decision, and 35.5% (n = 138) remaining neutral. Notably, physician recommendation had a strong impact, with 73.7% (n = 287) reporting willingness to receive the vaccine if advised by a doctor (Figure 2).

Participants’ attitudes toward HZ and vaccination varied significantly by gender, age, and chickenpox history. Regarding worry about getting HZ, a higher proportion of males (78; 64.5%) than females (126; 47.0%) were likely to be concerned, while a higher proportion of females (88; 32.8%) remained neutral compared to males (27; 22.3%) (p = 0.006). Respondents aged 50-59 years were the most likely to worry about HZ (74; 68.5%) compared to those aged 18-49 years (121; 45.7%) and ≥60 years (9; 56.3%) (p = 0.002). Among those with a history of chickenpox, 95 (44.0%) reported being concerned about HZ, while only 22 (16.1%) of those without a history reported the same (p < 0.001) (Table 4).

**Table 4:** Association between demographic variables and attitudes toward herpes zoster and its vaccination (n = 389) p-values were calculated using the Chi-square test for independence. Response categories were collapsed into three groups: unlikely (extremely unlikely and unlikely), neutral, and likely (likely and extremely likely). Percentages were calculated within each subgroup (e.g., gender, age group, or chickenpox history) based on subgroup totals; therefore, percentages are not directly comparable across different demographic categories.

Question	Category	Unlikely n (%)	Likely n (%)	Neutral n (%)	p-value
I am worried about getting shingles.	Male	16 (13.2)	78 (64.5)	27 (22.3)	0.006
	Female	54 (20.1)	126 (47.0)	88 (32.8)	
	18–49 years	56 (21.1)	121 (45.7)	88 (33.2)	0.002
	50–59 years	12 (11.1)	74 (68.5)	22 (20.4)	
	60+ years	2 (12.5)	9 (56.3)	5 (31.3)	
	Had chickenpox	40 (18.5)	95 (44.0)	81 (37.5)	<0.001
	No chickenpox	23 (16.8)	92 (67.2)	22 (16.1)	
	Don’t know	7 (19.4)	17 (47.2)	12 (33.3)	
I am interested in knowing more about this disease.	Male	4 (3.3)	66 (54.5)	51 (42.1)	<0.001
	Female	12 (4.5)	228 (85.1)	28 (10.4)	
	18–49 years	12 (4.5)	226 (85.3)	27 (10.2)	<0.001
	50–59 years	2 (1.9)	56 (51.9)	50 (46.3)	
	60+ years	2 (12.5)	12 (75.0)	2 (12.5)	
	Had chickenpox	6 (2.8)	179 (82.9)	31 (14.4)	<0.001
	No chickenpox	7 (5.1)	83 (60.6)	47 (34.3)	
	Don’t know	3 (8.3)	32 (88.9)	1 (2.8)	
I am interested in knowing more about how to prevent this disease.	Male	2 (1.7)	72 (59.5)	47 (38.8)	<0.001
	Female	11 (4.1)	245 (91.4)	12 (4.5)	
	18–49 years	11 (4.2)	242 (91.3)	12 (4.5)	<0.001
	50–59 years	2 (1.9)	60 (55.6)	46 (42.6)	
	60+ years	0 (0.0)	15 (93.8)	1 (6.3)	
	Had chickenpox	4 (1.9)	197 (91.2)	15 (6.9)	<0.001
	No chickenpox	6 (4.4)	88 (64.2)	43 (31.4)	
	Don’t know	3 (8.3)	32 (88.9)	1 (2.8)	
The vaccine cost will affect my decision to take the vaccine.	Male	18 (14.9)	37 (30.6)	66 (54.5)	<0.001
	Female	93 (34.7)	103 (38.4)	72 (26.9)	
	18–49 years	75 (28.3)	117 (44.2)	73 (27.5)	<0.001
	50–59 years	29 (26.9)	16 (14.8)	63 (58.3)	
	60+ years	7 (43.8)	7 (43.8)	2 (12.5)	
	Had chickenpox	64 (29.6)	89 (41.2)	63 (29.2)	0.007
	No chickenpox	32 (23.4)	42 (30.7)	63 (46.0)	
	Don’t know	15 (41.7)	9 (25.0)	12 (33.3)	
I would get the vaccine if the doctor recommended it.	Male	8 (6.6)	62 (51.2)	51 (42.1)	<0.001
	Female	12 (4.5)	225 (84.0)	31 (11.6)	
	18–49 years	15 (5.7)	218 (82.3)	32 (12.1)	<0.001
	50–59 years	3 (2.8)	55 (50.9)	50 (46.3)	
	60+ years	2 (12.5)	14 (87.5)	0 (0.0)	
	Had chickenpox	9 (4.2)	178 (82.4)	29 (13.4)	<0.001
	No chickenpox	6 (4.4)	81 (59.1)	50 (36.5)	
	Don’t know	5 (13.9)	28 (77.8)	3 (8.3)	

**Figure 3:**
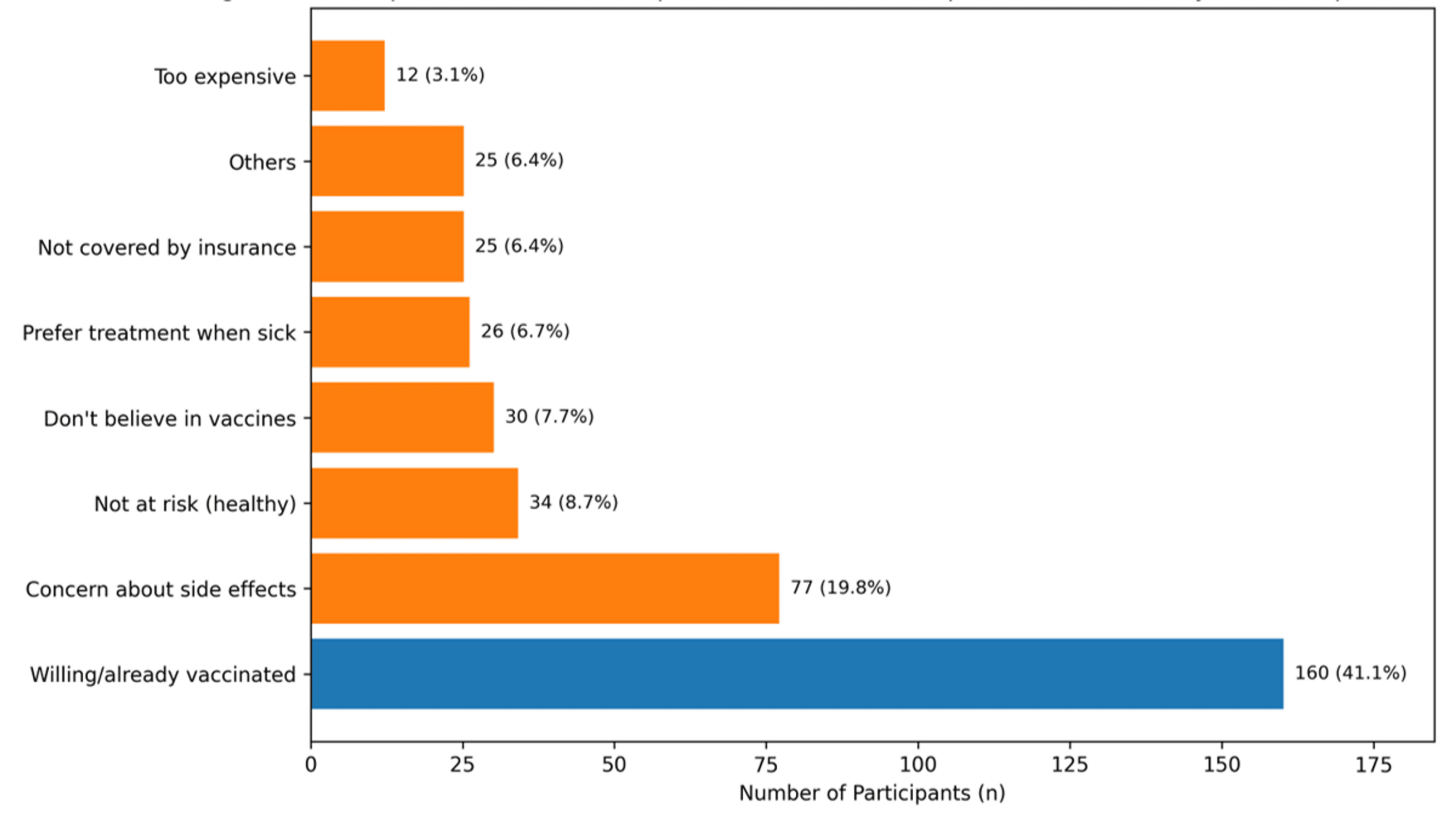
Participants’ reasons for herpes zoster vaccine acceptance and hesitancy (multi-response) (n = 389) Values are presented as n (%). The blue bar represents vaccine acceptance, and the orange bars represent reasons for hesitancy. Percentages are calculated from the total sample (n = 389); in multi-response, participants could select more than one option.

Figure 3 presents participants’ reasons for vaccine acceptance (blue) and hesitancy (orange). The most common response was willingness to receive the HZ vaccine or prior vaccination (41.1%; n = 160). Among the reasons for hesitancy, concerns about side effects were most frequently reported (19.8%; n = 77), followed by low perceived risk (8.7%; n = 34). Smaller proportions reported not believing in vaccines (7.7%; n = 30), preferring to take medication only when ill (6.7%; n = 26), lack of insurance coverage (6.4%; n = 25), other unspecified reasons (6.4%; n = 25), and cost concerns (3.1%; n = 12). Overall, 6.4% (n = 25) of participants reported having already received the HZ vaccine.

## Discussion

Our multi-center survey of adults in UAE primary care settings revealed generally moderate awareness of HZ and its vaccine, with a majority demonstrating some knowledge but relatively few with high knowledge. Specifically, only 17% of respondents had high knowledge scores, while 32% had low knowledge, and the rest were moderate. Our findings align with a previous study from the UAE by Al-Khalidi et al. [9]. Their findings also showed a mean knowledge score of HZ of 39.3%; however, only 1.9% of the participants in their study had a knowledge score above 80%. Similarly, in another study from Hong Kong, Lam et al. reported that only half of the respondents were able to answer five or six questions correctly, with a mean correct response of 4.96 (±1.72) per eight questions [12]. This level of awareness also broadly aligns with recent regional studies. For example, a 2024 Saudi nationwide survey similarly found that only about 7.8% of adults had high knowledge of HZ, and only 3.1% had adequate knowledge of its vaccine [10]. Another Saudi study of adults ≥50 years found a mean HZ knowledge score of 28.6% and vaccine knowledge of 37.1% [13]; only 31.6% of respondents achieved a knowledge score ≥80% on the HZ vaccine.

Our finding that higher education and prior chickenpox history predicted greater HZ knowledge is also consistent with prior reports. We observed a strong association between education level and knowledge (p < 0.001), as university and postgraduate attendees had substantially higher knowledge than high-school graduates. Higher education was associated with higher knowledge, consistent with health literacy literature. Al-Khalidi et al. found postgraduate UAE residents had significantly higher HZ knowledge scores [9], which mirrors global trends that education correlates with health literacy. Likewise, we found participants with a history of chickenpox more often had moderate-to-high knowledge (18.1% high vs 17.5% among those without chickenpox), consistent with Alhothali et al., who also noted prior varicella as a predictor of HZ knowledge [14]. Internationally, a Hong Kong survey similarly reported that a history of HZ/varicella infection and higher education significantly boosted knowledge score [12]. Together, these results suggest that personal experience with VZV or higher education likely raises awareness.

Furthermore, in the present study, we also identified that male gender and older age were associated with poorer knowledge. Lam et al. also reported that HZ knowledge was negatively correlated with male gender (β = -0.396, p = 0.029) [12]. Similarly, another study by Xiaoyuan et al. from China reported that male gender was negatively correlated with HZ knowledge (β = -0.51, p < 0.001) [15]. Al-Khalidi found being female to be a positive predictor of HZ awareness in the UAE [9]. In our data, older participants were more likely to have low knowledge, perhaps reflecting differences in formal education or health information access across age cohorts. Despite these knowledge gaps, attitudes toward HZ prevention were generally favorable. A large majority of respondents (over 75%) expressed interest in learning more about HZ and its prevention, and about 52% were worried or likely to worry about getting HZ. Furthermore, 73.8% said they would take the vaccine if recommended by a doctor.

These figures are consistent with a broadly positive public attitude observed in recent studies, even where actual vaccine uptake remains low. For example, Alhothali et al. reported that 77.4% of Saudi adults over 50 years would accept the HZ vaccine if a healthcare professional recommended it [14], and our findings (74%) are very similar. Likewise, a large-scale review found that in WHO countries, 75.19% of older adults are willing to vaccinate, particularly if endorsed by providers [16]. The critical role of physicians is underscored by multiple sources. Similarly, Al-Khalidi’s study found an odds ratio of 26 for HZ vaccination uptake on doctor recommendation [9]. Globally, only about half of older adults express willingness to vaccinate on their own (56% without prompting), but willingness jumps to 75% with a provider’s recommendation [16]. Nonetheless, we also identified important attitudinal barriers. Over one-third of participants (28.5%) said cost would affect their decision, and 35% were neutral, indicating that financial concern is a real factor. This resonates with international reports: cost and financial concerns consistently emerge as reasons for hesitancy or non-uptake [17]. Beyond financial concerns, our findings also revealed other prominent barriers, including fear of side effects, low perceived susceptibility, and, to a lesser extent, distrust in vaccines. Similar patterns have been reported in previous studies, where concerns about vaccine safety and a perceived low personal risk were among the most frequently cited reasons for hesitancy toward HZ and other adult vaccines [9,14]. Addressing these attitudinal and structural factors through targeted education, physician engagement, and policy measures is essential to ensure that high willingness to vaccinate is translated into actual uptake.

We also observed intriguing gender and age differences in attitudes. Women in our study were significantly more interested than men in both learning about HZ (85% vs 55% “likely” interested) and knowing how to prevent it (91% vs 60%). Conversely, men (30.6%) were more likely than women (26.9%) to say vaccine cost would affect them. Our survey found that prior varicella (chickenpox) infection was associated with more interest and uptake intentions toward the HZ vaccine. Those with a history of chickenpox were significantly more likely to express interest in HZ prevention (91.2% vs 64.2%) and willingness to vaccinate on doctor recommendation (82.4% vs 59.1%) than those without. Interestingly, participants unaware of their varicella history were even more likely to say cost would affect them (41.7%) than those who had or had not had chickenpox, suggesting uncertainty contributes to hesitancy.

When contextualizing our findings with the regional literature, a consistent theme emerges: knowledge about HZ and its vaccine is generally inadequate, while attitudes (especially willingness under physician influence) are positive but uptake is poor. The implications of our findings are clear. Educational efforts must be stepped up. Broad public health campaigns, similar to those called for in recent Saudi and UAE studies, could significantly increase awareness of the recombinant HZ vaccine and its benefits. Given that higher education strongly predicts knowledge, materials should be tailored to reach lower-educated groups via simple messages. Because physician recommendations are such a powerful motivator, healthcare providers in UAE clinics should proactively discuss HZ prevention with eligible patients.

These findings have important implications for the UAE’s adult vaccination program. Strengthening targeted awareness campaigns for demographic groups with lower knowledge, such as males and older adults, could improve vaccine uptake. Policy measures, including integrating HZ vaccination into existing adult immunization schedules and subsidizing vaccine costs for high-risk groups, may further enhance coverage. Comparisons with uptake of other adult vaccines in the UAE, such as influenza and pneumococcal vaccines, indicate similar gaps in awareness and uptake, suggesting that multi-vaccine education strategies could be beneficial. The findings of this study align closely with the broader goals of the UAE Vision 2031, which emphasizes enhancing community health literacy, advancing preventive healthcare, and strengthening digital innovation across the health sector. The moderate awareness but high willingness to vaccinate observed among UAE adults reflects an opportunity for targeted, technology-driven educational interventions that resonate strongly with Vision 2031’s public health and digital transformation pillars. Vision 2031 prioritizes disease prevention, health innovation, and continuous professional education to build a proactive, digitally empowered healthcare system. The digital continuing medical education (CME) shift underway in the UAE provides a strong platform to address these gaps [18]. The use of virtual CME modules and AI-driven learning tools can keep physicians informed on immunization practices. Incorporating HZ vaccination content into these programs could enhance physicians’ confidence in recommending vaccines.

Limitations

This study has several limitations that should be considered when interpreting the findings. First, its cross-sectional design captures associations at a single point in time and cannot establish causality between demographic characteristics, knowledge, attitudes, and vaccination practices. Second, the use of a self-administered questionnaire, while practical for reaching a large sample, may have introduced recall bias, social desirability bias, and potential satisficing due to mandatory response fields, potentially leading to over- or underestimation of knowledge and attitudes. Internal consistency of the adapted instrument was not reassessed following modification, which may affect the reliability of the findings. Third, although the sample size was adequate for statistical analysis, the disproportionately high participation of females and Emirati nationals may limit the generalizability of results to other demographic groups. Because recruitment occurred in EHS primary healthcare centers, Emiratis and insured individuals, who are more likely to utilize these services, were overrepresented.

Nationality was self-reported, and while expatriates were included, their representation may not reflect their distribution in the general population, as many may seek care in private or non-EHS facilities. Therefore, the findings may not be fully generalizable to expatriate populations. Fourth, the survey was conducted among attendees of primary healthcare centers, who may have higher baseline health awareness compared to the general population, potentially inflating knowledge scores. Additionally, the use of a non-probability convenience sampling method, while practical for rapid multi-center recruitment and consistent with prior UAE-based HZ studies, may introduce selection bias and further limit the generalizability of findings, as it likely overrepresents individuals who actively attend PHC centers and may have higher baseline health awareness. Recruitment was not proportional across emirates, which may introduce cluster and catchment-area effects and further limit the generalizability of findings to the national population.

Additionally, some subgroup analyses involved small sample sizes (e.g., older age groups and lower education levels), which may result in unstable estimates and should be interpreted with caution. Multivariable analysis was not performed, as the study was designed as an exploratory analysis primarily aimed at identifying associations using bivariate methods. Response rate data (number invited versus completed) were not available from the EHS Data Hub system. Finally, the study did not include a longitudinal follow-up to assess whether positive attitudes translated into actual vaccination uptake, which would be an important next step for future research.

## Conclusions

This study highlights current levels of knowledge and attitudes toward herpes zoster (HZ) and its vaccine among adults attending primary healthcare centers in the UAE. While many participants demonstrated moderate knowledge, notable gaps remain, particularly among males, individuals aged 50-59 years, and those with lower educational levels. Awareness of the disease and its prevention was not consistent, which may affect timely vaccination. Despite these gaps, attitudes toward HZ prevention were generally positive. Most participants expressed interest in learning more and indicated a willingness to receive the vaccine, especially when recommended by healthcare professionals.

Further research is needed to better understand barriers to vaccine access and uptake, and longitudinal studies could help track changes over time. Strengthening public education and encouraging physicians to initiate vaccination discussions may help bridge the gap between awareness and actual vaccine uptake.

## Appendices

Appendix 1: Questionnaire

Title: Knowledge, attitudes, and practices toward herpes zoster and its vaccine among primary health care attendees in the United Arab Emirates: a cross-sectional study

Section 1: Sociodemographic Information

Age:

☐ 18-49

☐ 50-59

☐ ≥60

Gender:

☐ Male

☐ Female

Nationality:

☐ Emirati

☐ Arab (non-Emirati)

☐ GCC

☐ Other

Employment Status:

☐ Employed

☐ Unemployed

☐ Retired

☐ Self-employed

Education Level:

☐ Less than high school

☐ High school

☐ University/College

☐ Postgraduate

Do you have health insurance?

☐ Yes

☐ No

Have you ever had chickenpox?

☐ Yes

☐ No

☐ Don’t know

Do you have any chronic disease? (Select all that apply)

☐ Hypertension

☐ Diabetes

☐ Thyroid disease

☐ COPD

☐ Autoimmune disease

☐ Cardiovascular disease

☐ Kidney disease

☐ Cancer

☐ None

Section 2: Knowledge About Herpes Zoster and Vaccine

Have you heard of herpes zoster (shingles)?

☐ Yes

☐ No

What causes herpes zoster?

☐ Virus

☐ Bacteria

☐ Don’t know

Who is at higher risk of developing shingles?

☐ Older adults

☐ Children

☐ Don’t know

Can herpes zoster cause complications?

☐ Yes

☐ No

☐ Don’t know

Have you heard about the herpes zoster vaccine?

☐ Yes

☐ No

Who should receive the herpes zoster vaccine?

☐ Adults ≥50 years

☐ Children

☐ Don’t know

Can the vaccine prevent shingles?

☐ Yes

☐ No

☐ Don’t know

Is the vaccine available in the UAE?

☐ Yes

☐ No

☐ Don’t know

Is herpes zoster contagious?

☐ Yes

☐ No

☐ Don’t know

Can someone get shingles more than once?

☐ Yes

☐ No

☐ Don’t know

Section 3: Attitudes Toward Herpes Zoster and Vaccination

(Likert scale: 1 = Very unlikely, 2 = Unlikely, 3 = Neutral, 4 = Likely, 5 = Extremely likely)

19.  I am worried about getting shingles.

20.  I am interested in knowing more about this disease.

21.  I am interested in knowing more about how to prevent this disease.

22.  The vaccine cost will affect my decision to take the vaccine.

23.  I would get the vaccine if a doctor recommended it.

1 = Very unlikely

2 = Unlikely

3 = Neutral

4 = Likely

5 = Extremely likely

19.       I am worried about getting shingles.

20.       I am interested in knowing more about this disease.

21.       I am interested in knowing more about how to prevent this disease.

22.       The vaccine cost will affect my decision to take the vaccine.

23.       I would get the vaccine if a doctor recommended it.

Section 4: Practices Toward Herpes Zoster and Vaccination

Have you received the herpes zoster vaccine?

☐ Yes

☐ No

If no, what is the reason? (Select all that apply)

☐ Concern about side effects

☐ Do not believe in vaccines

☐ Not at risk (healthy)

☐ Prefer treatment only when sick

☐ Not covered by insurance

☐ Too expensive

☐ Other: ______

العنوان: المعرفة والمواقف والممارسات تجاه الهربس النطاقي (الحزام الناري) والتطعيم الخاص به بين مرتادي مراكز الرعاية الصحية الأولية في دولة الإمارات العربية المتحدة

القسم الأول: المعلومات الديموغرافية

العمر:

☐ 18-49 سنة

☐ 50-59 سنة

☐ 60 سنة فأكثر

الجنس:

☐ ذكر

☐ أنثى

الجنسية:

☐ إماراتي

☐ عربي (غير إماراتي)

☐ خليجي

☐ أخرى

الحالة الوظيفية:

☐ موظف

☐ غير موظف

☐ متقاعد

☐ عمل حر

المستوى التعليمي:

☐ أقل من الثانوية

☐ الثانوية العامة

☐ جامعي

☐ دراسات عليا

هل لديك تأمين صحي؟

☐ نعم

☐ لا

هل سبق أن أُصبت بجدري الماء؟

☐ نعم

☐ لا

☐ لا أعلم

هل تعاني من أمراض مزمنة؟ (يمكن اختيار أكثر من خيار)

☐ ارتفاع ضغط الدم

☐ السكري

☐ أمراض الغدة الدرقية

☐ أمراض الرئة المزمنة (COPD)

☐ أمراض مناعية

☐ أمراض القلب

☐ أمراض الكلى

☐ السرطان

☐ لا يوجد

القسم الثاني: المعرفة حول الهربس النطاقي والتطعيم

هل سمعت من قبل عن الهربس النطاقي (الحزام الناري)؟

☐ نعم

☐ لا

ما سبب الهربس النطاقي؟

☐ فيروس

☐ بكتيريا

☐ لا أعلم

من الأكثر عرضة للإصابة بالحزام الناري؟

☐ كبار السن

☐ الأطفال

☐ لا أعلم

هل يمكن أن يسبب الحزام الناري مضاعفات؟

☐ نعم

☐ لا

☐ لا أعلم

هل سمعت عن لقاح الحزام الناري؟

☐ نعم

☐ لا

من الفئة التي يُنصح لها بأخذ اللقاح؟

☐ البالغون ≥50 سنة

☐ الأطفال

☐ لا أعلم

هل يمكن للقاح أن يمنع الإصابة بالحزام الناري؟

☐ نعم

☐ لا

☐ لا أعلم

هل اللقاح متوفر في دولة الإمارات؟

☐ نعم

☐ لا

☐ لا أعلم

هل الحزام الناري مرض معدٍ؟

☐ نعم

☐ لا

☐ لا أعلم

هل يمكن أن يُصاب الشخص بالحزام الناري أكثر من مرة؟

☐ نعم

☐ لا

☐ لا أعلم

القسم الثالث: المواقف تجاه الحزام الناري والتطعيم

(مقياس ليكرت: 1 = غير محتمل جدًا، 2 = غير محتمل، 3 = محايد، 4 = محتمل، 5 = محتمل جدًا)

1 = غير محتمل جدًا

2 = غير محتمل

3 = محايد

4 = محتمل

5 = محتمل جدًا

19.       أشعر بالقلق من الإصابة بالحزام الناري.

20.       أرغب في معرفة المزيد عن هذا المرض.

21.       أرغب في معرفة كيفية الوقاية من هذا المرض.

22.       تكلفة اللقاح تؤثر على قراري بأخذه.

23.       سأأخذ اللقاح إذا أوصى به الطبيب.

القسم الرابع: الممارسات تجاه الحزام الناري والتطعيم

هل سبق أن أخذت لقاح الحزام الناري؟

☐ نعم

☐ لا

إذا كانت الإجابة لا، ما السبب؟ (يمكن اختيار أكثر من خيار)

☐ الخوف من الآثار الجانبية

☐ لا أؤمن باللقاحات

☐ أعتقد أنني غير معرض للخطر

☐ أفضل العلاج عند المرض فقط

☐ غير مشمول بالتأمين

☐ مكلف جدًا

☐ أسباب أخرى: ______
